# The association between continuous polypharmacy and hospitalization, emergency department visits, and death in older adults: a nationwide large cohort study

**DOI:** 10.3389/fphar.2024.1382990

**Published:** 2024-07-31

**Authors:** Jungmi Chae, Ho Jin Cho, Sang-Heon Yoon, Dong-Sook Kim

**Affiliations:** ^1^ Department of Research, Health Insurance Review and Assessment Service, Wonju, Republic of Korea; ^2^ Department of Health Administration, Kongju National University, Gongju, Republic of Korea

**Keywords:** polypharmacy, elderly, outpatients, hospitalization, emergency department visit, death

## Abstract

**Background:**

This study aimed to investigate the association between continuous polypharmacy and hospitalization, emergency department (ED) visits, and death.

**Methods:**

This retrospective study utilized 6,443,896 patients aged between 65 and 84 years of National Health Insurance claims data from 2016 to 2018. Polypharmacy and excessive polypharmacy were defined as the concurrent use of 5 or more and 10 or more medications, respectively, for durations of both 90 days or more and 180 days or more within a 1-year observation period. The primary outcome measures included all-cause hospitalization, ED visits, and mortality. Multiple logistic regression models were used adjusting for patients’ general characteristics, comorbidities, and history of hospitalization or ED visits.

**Results:**

Among 2,693,897 patients aged 65–84 years who had used medicines for 180 days or more (2,955,755 patients taking medicines for 90 days or more), the adverse outcomes were as follows: 20.5% (20.3%) experienced hospitalization, 10.9% (10.8%) visited the ED, and 1% (1%) died, respectively. In patients who exhibited polypharmacy for more than 180 days, the adjusted odds ratio of adverse outcomes was 1.32 (95% confidence interval [CI], 1.31–1.33) for hospitalization, 1.32 (95% CI, 1.31–1.33) for ED visits, 1.63 (95% CI, 1.59–1.67) for death, and that in excessive polypharmacy patients for more than 180 days was 1.85 for hospitalization, 1.92 for ED visits, and 2.57 for death, compared to non-polypharmacy patients.

**Conclusion:**

Our results suggest that polypharmacy in older adults might lead to negative health consequences. Thus, interventions to optimize polypharmacy may need to be implemented.

## Introduction

Due to population aging, the number of older adults with multiple morbidities is increasing (Barnett et al., 2012; Masnoon et al., 2017; Khezrian et al., 2020; Hsu et al., 2021). Although multimorbidity levels are rising, the growing use of single-disease guidelines and adherence to evidence-based practice could potentially raise the risk of polypharmacy (Smith et al., 2012; Stewart et al., 2017; Smith et al., 2021). Polypharmacy is the concurrent use of multiple medications, often defined by the World Health Organization as the regular use of 5 or more medications (WHO, 2019). Additionally, hyper-polypharmacy is commonly defined as the use of ten or more medications in numerous studies (Masnoon et al., 2017). Many studies have defined polypharmacy as the concurrent use of 5 or more medications (Khezrian et al., 2020; Pazan and Wehling, 2021); however, there is debate regarding whether this concept should be defined based on only the number of medications.

Conversely, it is acknowledged that polypharmacy may be necessary for patients with severe multimorbidity, depending on the clinical context (Cadogan et al., 2016). Also, it is a challenge to reduce potentially harmful multiple medicines defined as “problematic polypharmacy,” which needs to be differentiated as appropriate polypharmacy (Taghy et al., 2020; Lau et al., 2023). Despite the difficulties of definition, polypharmacy has been associated with an increased likelihood of potentially inappropriate prescribing, including drug-drug interactions and the use of high-risk medications (Maher et al., 2014; Payne, 2016). It may also lead to reduced adherence to treatment regimens (Sergi et al., 2011; Barnett et al., 2016). Since older individuals often experience diminished drug metabolism and renal excretion capabilities, they may be particularly susceptible to significant health issues resulting from polypharmacy (Klotz, 2009).

Over the past few decades, numerous studies have investigated the consequences of polypharmacy in older adults. Five previous systematic reviews examining the effects of polypharmacy on adverse health outcomes consistently found an increased risk of falls, hospitalization, inappropriate prescribing, and mortality. However, these reviews reported conflicting results regarding adverse drug events and disability (Leelakanok et al., 2003; Fried et al., 2014; Palmer et al., 2019; Davies et al., 2020; Li et al., 2022). Additionally, several other systematic reviews focused on specific diseases have explored the negative consequences of polypharmacy, such as all-cause mortality in conditions like diabetes mellitus, cancer, atrial fibrillation, and chronic renal failure (Al-Musawe et al., 2019; Gallagher et al., 2020; Mohamed et al., 2020; Chen et al., 2021; Okpechi et al., 2021). Nevertheless, only a few studies have concurrently examined falls, hospitalization, and emergency department (ED) visits as indicators of health outcomes (Matsuyama et al., 2021; Suh et al., 2021; Turnbull et al., 2021; Zakarias et al., 2021; Nightingale et al., 2022; Perreault et al., 2022; van Dam et al., 2022; Wang et al., 2022; Fahmi et al., 2023; Liang et al., 2023). While two studies have simultaneously investigated the risk of hospitalization and death, their methodologies varied significantly. One study defined polypharmacy as the intake of several medications for at least 1 day, while the other focused on a population of patients following ED visits (Salvi et al., 2017; Chang et al., 2020).

Most previous observational studies have defined polypharmacy as the use of 5 or more medications, regardless of duration, and have compared these patients to the general population. This approach may introduce bias by comparing individuals on multiple medications with healthy individuals. Also, in clinical settings, patients with chronic diseases who require long-term prescriptions for chronic diseases may sometimes need to use 5 or 10 or more medicines due to an acute disease, so there is a need for evidence to generate the long-term effects of polypharmacy on health.

This study aimed to investigate the association between polypharmacy in older adults and adverse health outcomes, including hospitalization, ED visits, and death, compared to non-polypharmacy despite continuous medicine taking in South Korea. We considered the medication period when defining polypharmacy and examined the association between taking 5 or more or 10 or more medications for periods exceeding 90 and 180 days, respectively, and the subsequent adverse outcomes.

## Methods

This study received review and approval from the Institution Review Board of HIRA and patient consent was not required because anonymous retrospective data were used.

### Data sources and study population

We conducted a retrospective cohort study on older adults using National Health Insurance (NHI) claims data from January 2016 to December 2018, which was organized by the month of medical treatment provided. In Korea, the NHI covers 97% of the population, and the claims data are submitted by medical institutions to the Health Insurance Review and Assessment Service (HIRA) for payment. This NHI claims data set includes patient demographic characteristics, diagnosis codes for diseases, the international non-proprietary names of prescribed drugs, the daily dosage, and the duration of therapy (Lee et al., 2021).

The study population comprised older adults between the ages of 65 and 84, and their outpatient medical data were utilized. The medical institutions considered in the study were restricted to tertiary hospitals, secondary hospitals, general hospitals (which encompass healthcare facilities), clinics, and public health centers. Parenteral medicines were excluded from the analysis.

### Definition of polypharmacy and exposure periods

Polypharmacy was defined as the prescription of 5 or more drugs for 90 or 180 or more days, and hyper-polypharmacy was defined as the prescription of 10 or more drugs within the 1-year observation period.

To establish a cohort, we defined each patient’s index date as the day of the first outpatient visit for patients who received at least one outpatient prescription between January and December 2017. We then defined the exposure period as 1 year from the index date and the history as 1 year before the index date. Follow-up was conducted for 1 year after the end of the exposure period.

The year following each patient’s initial visit in 2017 was designated as the polypharmacy exposure period, while the subsequent year served as the health performance observation period. Patient data spanning from 2017 to 2019 were subjected to analysis, adhering to uniform criteria for both the exposure and observation periods, which consisted of 1 year each. The requirement of 2 years of data for each patient necessitated the use of the 2017 cohort to eliminate any potential confounding effects due to the coronavirus disease 2019 pandemic in 2020.

**FIGURE 1:**
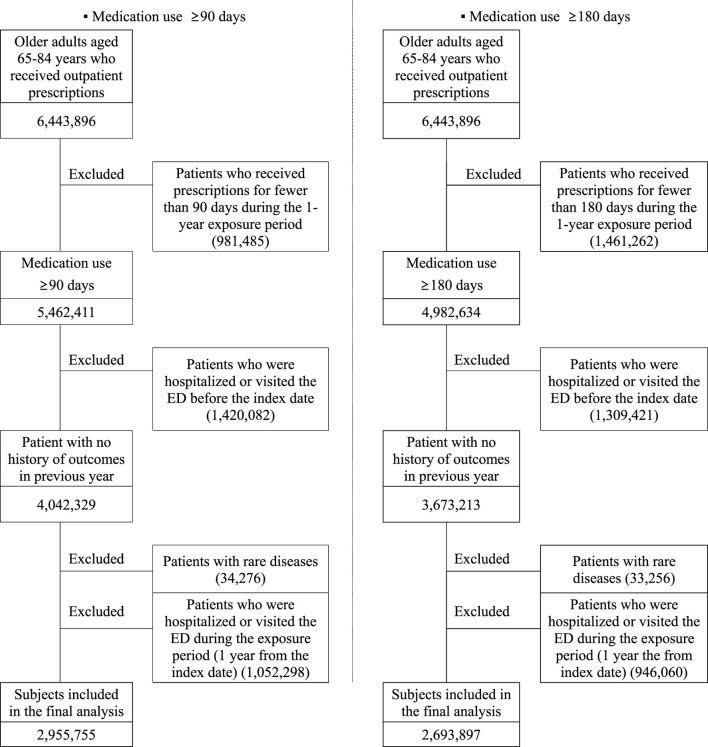
Study population selection for the analysis of the correlation between polypharmacy and health outcomes.

As shown in Figure 1, the selection of the study population involved excluding patients who received prescriptions for fewer than 90 days (or 180 days). Furthermore, to mitigate the impact of other variables on health outcomes, patients who had been hospitalized or had ED visits within 1 year prior to their first outpatient visit in 2017 were also excluded, as were patients with a rare disease.

### Outcome measures and confounding factors

The health outcomes included in this study were hospitalization, ED visits, and mortality. We examined a range of independent variables, including general status (categorized by sex, age, and insurance type), comorbidity status (encompassing 12 specific diseases [cancer, hypertension, hyperlipidemia, diabetes, cardio-vascular disease, stomach ulcer, chronic renal disease, liver disease, respiratory system disease, musculoskeletal disease, bone fracture, dementia], the total number of comorbidities, and classification according to the Elixhauser Comorbidity Index [ECI]), and utilization of medical services (quantified by the number of institutions issuing prescriptions, the number of departments issuing prescriptions, and the total number of outpatient visits).

### Statistical analysis

To investigate the impact of polypharmacy on health outcomes, we performed a simple logistic regression analysis for each variable. Additionally, a multinomial logistic regression analysis was conducted. Model 1 was adjusted for age, sex, and insurance type to analyze the correlation between health outcomes and polypharmacy. Model 2 was further adjusted for the number of comorbidities and the ECI. Model 3 included all the aforementioned variables and also controlled for the number of outpatient visits. SAS Enterprise version 7.1 (SAS Institute, Cary, NC, United States) was used for all analyses.

## Results

### General characteristics

Among older adults who took medications for 90 or more days, 37.8% were classified as polypharmacy patients, and 8.0% were considered hyper-polypharmacy patients. In the group of older adults who took medications for 180 or more days, the figures were 32.8% for polypharmacy and 5.1% for hyper-polypharmacy. Among the elderly who use medication for ≥180 days, males accounted for 42.4% in the non-polypharmacy group, whereas 43.8% were in the polypharmacy group and 45.7% were in the hyper-polypharmacy group. Also, the polypharmacy and hyper-polypharmacy groups compared to the non-polypharmacy group, showed a higher percentage of patients eligible for medical aid and veterans. The age group of 65–69 years had the highest proportion of polypharmacy patients, while hyper-polypharmacy was most prevalent in the 75–79-year age bracket. A significant number of patients suffered from hypertension, musculoskeletal disorders, hyperlipidemia, and diabetes. Polypharmacy was frequently observed in patients with these comorbidities, with the exception of those with hyperlipidemia and liver disease. Additionally, a higher number of comorbidities and underlying diseases was higher in polypharmacy and hyper-polypharmacy (Table 1).

**TABLE 1:** General characteristics of the cohort of older adults (%).

No. of patients (1,000 patients)	Medication use ≥90 days				Medication use ≥180 days			
	Total (N = 2,955,755)	Non-polypharmacy (N = 1,601,882)	Polypharmacy (N = 1,116.875)	Hyper-polypharmacy (N = 236,998)	Total (N = 2,693,987)	Non-polypharmacy (N = 1,673,910)	Polypharmacy (N = 882,555)	Hyper-polypharmacy (N = 137,432)
Sex								
Male	43	43.3	42.5	43.9	43	42.4	43.8	45.7
Female	57	56.7	57.5	56.1	57	57.6	56.2	54.3
Insurance type								
Health insurance	94.4	96.5	93.2	85.5	94.2	96.1	92.3	83.6
Medical Aid	5.6	3.5	6.7	14.3	5.7	3.8	7.6	16
Veteran	0.1	0	0.1	0.3	0.1	0	0.1	0.4
Age								
65–69 years	37.1	42.6	31.9	24.6	36.5	40.9	30.1	23.5
70–74 years	27.2	27.4	27.1	26.9	27.3	27.6	26.8	26.7
75–79 years	22.8	19.9	25.5	29.3	23.1	20.8	26.5	29.6
80–84 years	12.9	10.2	15.5	19.2	13.2	10.8	16.6	20.2
Mean ± SD, years	72.4 ± 5.4	71.6 ± 5.2	73.1 ± 5.5	74.0 ± 5.4	72.5 ± 5.4	71.8 ± 5.3	73.3 ± 5.5	74.2 ± 5.4
Comorbidities								
Cancer	5.9	5.7	5.9	7	6	5.9	6.1	7.1
Hypertension	59	53.8	64.5	68.2	63	61	66.3	67
Hyperlipidemia	35.6	34.8	36.6	36	37.2	38.2	35.6	34.7
Diabetes	27.7	17.9	36.8	51.4	29.6	20.7	42.6	54.7
Cardio-cerebrovascular disease	10	5.3	14.3	22	10.7	6.3	17.1	24.4
Stomach ulcer/GERD	13.1	11.2	14.7	19	12.9	12	13.9	17.3
Chronic renal disease	0.3	0.2	0.5	0.9	0.4	0.2	0.5	1.1
Liver disease	9.2	9	9.1	11	9.3	9.4	8.9	10.9
Respiratory system disease	11.6	9.4	13.2	19.5	11.6	10.2	13.1	18.9
Musculoskeletal disease	41.6	37.1	45.4	55.1	41.2	39.1	43.6	52.1
Bone fracture	3.8	3.3	4.1	5.6	3.7	3.4	4.1	5.7
Dementia	7.6	5.2	9.3	15.9	7.8	5.8	10.1	17.7
Number of comorbidities								
0	5.4	7.7	3.1	1.2	4.2	5.3	2.5	1.3
1	22.4	29.2	15.6	8.8	20.8	25.1	14.5	9.1
2	33.6	35.7	32.7	23.6	34.3	36.1	32.4	23.7
≥3	38.5	27.4	48.6	66.4	40.8	33.5	50.7	66
ECI								
0	13	18	7.9	3.1	10.5	13.2	6.6	3
1	34.8	42.2	28.1	16.3	34.5	40.7	25.8	15.5
2	30.1	27.4	34.1	29.1	31.3	30	34.3	28.3
≥3	22.2	12.4	30	51.5	23.7	16.2	33.4	53.1
Number of institutions								
1	7.8	9.2	6.4	4.1	7.9	8.6	7	5.3
2–3	29.3	32.5	26.5	21	29.6	31	27.8	24.1
4–5	29.2	30	28.7	26.6	29	29.4	28.6	27.3
≥6	33.7	28.3	38.4	48.4	33.4	30.9	36.7	43.3
Number of outpatient visits (days)								
1–10	11.9	16.6	7.3	1.8	11.1	13.5	7.8	2.1
11–30	54.1	62.4	47.8	26.9	53.8	59.1	47.8	29.1
31–50	21.1	15.6	27.1	30.4	21.5	18.9	25.5	28.9
>50	12.9	5.4	17.8	40.9	13.6	8.6	19	39.9

ECI, elixhauser comorbidity index; GERD, gastroesophageal reflux disease; SD, standard deviation.

### Adverse health outcomes

As shown in Table 2, 20.3% of seniors who were on medication for 90 or more days during the exposure period were hospitalized during the observation period. Additionally, 10.8% visited the ED, and 1.0% died. For those who were prescribed medications for 180 days or longer, the rates were slightly higher: 20.5% were hospitalized, 10.9% visited the ED, and 1.0% died. Notably, hyper-polypharmacy was more prevalent than polypharmacy among the individuals who were hospitalized, those who visited the ED, and those who died.

**TABLE 2:** The risk of adverse outcomes in older adults according to polypharmacy status [1,000 patients (%)].

Category	Number of patients		Non-polypharmacy		Polypharmacy		Hyper-polypharmacy		*p*-value
Medication use ≥90 days	2,956		1,602 (54.2)		1,117 (37.8)		237 (8.0)		<.0001
All-cause hospitalization	602	(20.3)	272	(17.0)	256	(22.9)	74	(31.2)	<.0001
Emergency department visits	318	(10.8)	141	(8.8)	135	(12.1)	42	(17.7)	<.0001
Death	29	(1.0)	11	(0.7)	13	(1.2)	5	(2.1)	<.0001
Medication use ≥180 days	2,694		1,674 (62.1)		883 (32.8)		137 (5.1)		<.0001
All-cause hospitalization	553	(20.5)	298	(17.8)	211	(23.9)	44	(32.1)	<.0001
Emergency department visits	293	(10.9)	153	(9.1)	114	(12.9)	26	(19.0)	<.0001
Death	27	(1.0)	12	(0.7)	12	(1.4)	3	(2.2)	<.0001

### The association between polypharmacy and health outcomes

**FIGURE 2:**
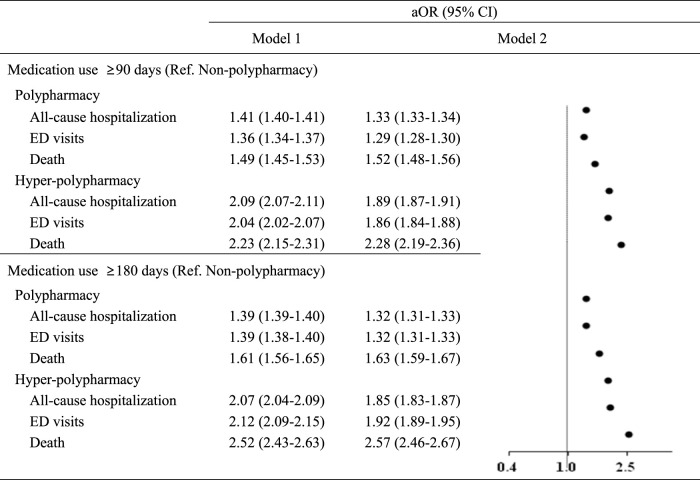
Associations between polypharmacy and adverse health outcomes ED, emergency department; aOR, adjusted odds ratio; CI, confidence interval. * Model 1: Controlled for sex, age, and insurance type.

As shown in Figure 2, in Model 1, which controlled for demographic information (sex and age) and insurance type, significant effects were noted. Patients with polypharmacy were 1.36–1.41 times more likely to be hospitalized or to visit the emergency room, and 1.49 to 1.61 times more likely to die compared to patients without polypharmacy. Those with hyper-polypharmacy were 2.04–2.12 times more likely to be hospitalized or to have emergency department visits, and 2.23 to 2.52 times more likely to die than their non-polypharmacy counterparts. In model 2, which controlled for the number of comorbidities and the ECI in addition to the variables from Model 1, the likelihood changed slightly. Polypharmacy patients were 1.29–1.33 times more likely to be hospitalized or to visit the emergency room, and 1.52 to 1.63 times more likely to die than those without polypharmacy. Hyper-polypharmacy patients were 2.28–2.57 times more likely to die than non-polypharmacy patients.

### Subgroup analysis

To exclude the effect of comorbidities on polypharmacy, a subgroup analysis was conducted for individuals both with and without comorbidities. As shown Table 3, the analysis revealed that, after adjusting for all significant variables, patients in both the polypharmacy and hyper-polypharmacy categories—regardless of comorbidity status—were 1.2 and 1.5 times more likely, respectively, to be hospitalized, to visit the ED, or to die compared to those not experiencing polypharmacy. Consequently, it was determined that polypharmacy significantly increases the risk of hospitalization, emergency room visits, and mortality in older adults, independent of comorbidities.

**TABLE 3:** Associations between polypharmacy and adverse health outcomes according to comorbidities.

	aOR (95% CI) in medication use ≥90 days			aOR (95% CI) in medication use ≥180 days		
	All-cause hospitalization	ED visits	Death	All-cause hospitalization	ED visits	Death
Cancer						
Polypharmacy	1.18 (1.15–1.22)	1.21 (1.17–1.25)	1.26 (1.15–1.39)	1.19 (1.16–1.22)	1.21 (1.17–1.25)	1.18 (1.15–1.21)
Hyper-polypharmacy	1.50 (1.44–1.56)	1.61 (1.53–1.69)	1.58 (1.38–1.80)	1.53 (1.46–1.61)	1.65 (1.56–1.74)	1.51 (1.45–1.59)
Hypertension						
Polypharmacy	1.25 (1.24–1.26)	1.24 (1.23–1.25)	1.23 (1.22–1.24)	1.24 (1.23–1.26)	1.26 (1.25–1.28)	1.23 (1.22–1.24)
Hyper-polypharmacy	1.63 (1.61–1.65)	1.70 (1.67–1.73)	1.59 (1.57–1.61)	1.61 (1.59–1.64)	1.75 (1.71–1.78)	1.59 (1.56–1.61)
Hyperlipidemia						
Polypharmacy	1.25 (1.23–1.26)	1.26 (1.24–1.28)	1.23 (1.22–1.24)	1.25 (1.23–1.26)	1.30 (1.28–1.32)	1.24 (1.22–1.25)
Hyper-polypharmacy	1.64 (1.61–1.67)	1.78 (1.74–1.82)	1.61 (1.58–1.63)	1.63 (1.60–1.67)	1.86 (1.81–1.91)	1.61 (1.58–1.65)
Diabetes						
Polypharmacy	1.22 (1.21–1.24)	1.22 (1.20–1.24)	1.20 (1.19–1.22)	1.23 (1.22–1.25)	1.24 (1.22–1.26)	1.21 (1.20–1.23)
Hyper-polypharmacy	1.64 (1.61–1.67)	1.73 (1.69–1.76)	1.60 (1.58–1.63)	1.65 (1.62–1.68)	1.78 (1.74–1.82)	1.62 (1.59–1.65)
Cardio-cerebrovascular disease						
Polypharmacy	1.20 (1.17–1.22)	1.22 (1.19–1.26)	1.18 (1.16–1.20)	1.21 (1.19–1.24)	1.24 (1.21–1.27)	1.20 (1.17–1.22)
Hyper-polypharmacy	1.56 (1.52–1.60)	1.64 (1.59–1.70)	1.53 (1.49–1.57)	1.55 (1.51–1.60)	1.67 (1.61–1.73)	1.53 (1.48–1.57)
Gastric ulcer/GERD						
Polypharmacy	1.18 (1.16–1.21)	1.17 (1.14–1.20)	1.17 (1.15–1.19)	1.22 (1.19–1.24)	1.24 (1.21–1.27)	1.20 (1.18–1.22)
Hyper-polypharmacy	1.53 (1.49–1.57)	1.60 (1.55–1.65)	1.49 (1.46–1.53)	1.55 (1.50–1.60)	1.70 (1.64–1.77)	1.52 (1.48–1.57)
Chronic renal failure						
Polypharmacy	1.32 (1.18–1.47)	1.49 (1.30–1.72)	1.32 (1.19–1.47)	1.33 (1.20–1.48)	1.48 (1.30–1.67)	1.34 (1.22–1.48)
Hyper-polypharmacy	1.80 (1.57–2.06)	1.90 (1.61–2.24)	1.77 (1.55–2.02)	1.74 (1.52–2.01)	1.83 (1.55–2.15)	1.71 (1.49–1.96)
Liver disease						
Polypharmacy	1.22 (1.20–1.25)	1.21 (1.18–1.24)	1.20 (1.17–1.22)	1.24 (1.21–1.27)	1.24 (1.20–1.27)	1.22 (1.20–1.25)
Hyper-polypharmacy	1.57 (1.51–1.62)	1.60 (1.54–1.67)	1.53 (1.49–1.58)	1.58 (1.52–1.64)	1.66 (1.58–1.74)	1.56 (1.50–1.62)
Respiratory disease						
Polypharmacy	1.23 (1.20–1.25)	1.25 (1.22–1.28)	1.21 (1.19–1.23)	1.24 (1.22–1.27)	1.28 (1.25–1.31)	1.23 (1.21–1.25)
Hyper-polypharmacy	1.57 (1.51–1.62)	1.72 (1.67–1.78)	1.58 (1.54–1.62)	1.60 (1.55–1.64)	1.75 (1.69–1.81)	1.58 (1.54–1.63)
Musculoskeletal disease						
Polypharmacy	1.22 (1.21–1.23)	1.20 (1.18–1.21)	1.20 (1.19–1.21)	1.22 (1.21–1.24)	1.23 (1.22–1.25)	1.21 (1.20–1.22)
Hyper-polypharmacy	1.56 (1.54–1.59)	1.63 (1.60–1.66)	1.53 (1.51–1.56)	1.58 (1.55–1.60)	1.70 (1.66–1.74)	1.55 (1.53–1.58)
Bone fracture						
Polypharmacy	1.29 (1.25–1.33)	1.23 (1.19–1.29)	1.25 (1.21–1.29)	1.28 (1.24–1.33)	1.31 (1.26–1.36)	1.26 (1.22–1.30)
Hyper-polypharmacy	1.73 (1.65–1.81)	1.80 (1.70–1.90)	1.68 (1.60–1.76)	1.67 (1.58–1.76)	1.87 (1.75–1.99)	1.62 (1.54–1.71)
Dementia						
Polypharmacy	1.24 (1.21–1.27)	1.30 (1.27–1.34)	1.23 (1.20–1.25)	1.24 (1.22–1.27)	1.32 (1.29–1.36)	1.24 (1.21–1.26)
Hyper-polypharmacy	1.63 (1.58–1.68)	1.81 (1.74–1.87)	1.61 (1.56–1.66)	1.62 (1.57–1.67)	1.81 (1.75–1.88)	1.59 (1.54–1.64)

ED, emergency department; GERD, gastroesophageal reflux disease; aOR, adjusted odds ratio; CI, confidence interval.

^*^Adjusting for sex, age, insurance type, the number of comorbidities, Elixhauser Comorbidity Index, and the number of outpatient visits.

## Discussion

In this extensive population-based cohort study of 2,955,755 older adults, we observed a significant association between polypharmacy and an increased risk of all-cause hospitalization, ED visits, and mortality. These results were consistent across definitions of polypharmacy, including being prescribed 5 or more medications for durations of 90 or 180 days or more, as well as hyper-polypharmacy, which was defined as being prescribed 10 or more medications. Specifically, patients experiencing polypharmacy and using medications for more than 180 days were 1.32 times more likely to be hospitalized or require ED visits and 1.63 times more likely to die compared to those not experiencing polypharmacy. For hyper-polypharmacy patients, the likelihood of mortality was 2.57 times greater than that of non-polypharmacy patients. Furthermore, we examined all outcomes in subgroup analysis according to comorbidity status and confirmed the association.

Although most previous studies on this topic have reported an association between polypharmacy and adverse health outcomes, there have been conflicting results regarding the risk of disability and mortality. Our results align with those of earlier studies, indicating that polypharmacy is associated with an increased risk of hospitalization and death. The adjusted hazard ratios (HRs) with 95% confidence intervals (CIs) were 1.16 (1.16–1.17) for hospitalization and 1.25 (1.24–1.25) for mortality in the matched cohorts, respectively (Chang et al., 2020). A UK study found that the number of medications was linked to higher hospitalization rates in Parkinson’s disease patients (HR = 1.23; 95% CI, 1.06–1.43) (Turnbull et al., 2021). In a Dutch study of ED patients aged 70 years and older, after adjusting for comorbidity, the odds ratio (OR) for mortality ranged from 1.80 (95% CI, 0.92–3.52) in patients with polypharmacy to 2.32 (95% CI, 1.10–4.90) in those with excessive polypharmacy (van Dam et al., 2022). An Italian study concluded that both polypharmacy and excessive polypharmacy were independent risk factors for adverse health outcomes—including in-hospital mortality, 30-day ED return, ED revisit, hospital admission, and 6-month mortality—following an ED visit (Salvi et al., 2017).

In our study, the males were found to be more likely to be polypharmacy and this phenomenon is consistent with two studies conducted in Korea (Kim et al., 2014; Cho et al., 2022). Many studies in other countries have reported that polypharmacy was higher in females (Zhang et al., 2020; Hellemans et al., 2021; Vos et al., 2022). This discrepancy could be due to medical culture in Korea and gender differences in attitude toward medication.

### Strengths and limitations

Our study has several noteworthy strengths. First, we examined the impact of polypharmacy on the risk of adverse outcomes within a nationwide population. Previous research has been limited by small sample sizes and a narrow range of outcomes. By analyzing National Health Insurance claims data that encompass the entire population, our findings are more broadly generalizable to other contexts, given the extensive scope of the data. Second, to the best of our knowledge, this is the largest population-based study to concurrently examine hospitalization, ED visits, and mortality associated with polypharmacy. Third, we categorized polypharmacy as the prescription of 5 or more drugs for either 90 or 180 days or longer, and hyper-polypharmacy as the prescription of 10 or more drugs within the 1-year observation period. In contrast, most previous studies have defined polypharmacy as the intake of 5 or more medications for at least 1 day. Regarding the comparator, we did not analyze a healthier population but rather comparable patients who had been taking medication for more than 90 or 180 days. Fourth, we used rigorous statistical methods to mitigate the risk of confounding by excluding patients who were hospitalized or had ED visits prior to the index date, as well as patients with rare diseases, in order to eliminate the influence of other potential underlying conditions on the analysis.

There are also several limitations to this study. It is retrospective in nature, and the lack of data on genetic factors, smoking, and lifestyle habits may limit its scope. Furthermore, we analyzed claims data and presumed that patients would take their prescribed medications. However, the prescribing records for some patients may not accurately reflect actual medication usage. Since this is an observational study, non-adherence to prescriptions could have impacted our results. Additionally, these databases do not account for over-the-counter or off-the-shelf medications taken by the patients. Especially, this study defined polypharmacy based on the numerical definition of medicines. Although there is appropriate polypharmacy, the point we could not distinguish the inappropriate polypharmacy is a limitation of this study. However, What is crucial is not polypharmacy but evaluating inappropriate polypharmacy to treat disease and promote patients’ health, and further research exploring unnecessary polypharmacy is warranted. We counted combination drugs as one drug, for example, a combination of beta-blockers and thiazide diuretics, and if a patient takes only three multiple compounds, he or she could be classified into the non-polypharmacy group. However, the elderly often visit hospitals for acute care, and these prescribing were added to routine chronic medication use. Also, assuming that the use of medicines may have been temporarily affected by the COVID-19 pandemic, a further study exploring the change of inappropriate polypharmacy before and after the COVID-19 pandemic will be needed.

Lastly, the current study has some of these limitations because of unmeasured factors, thereby our results on the association between polypharmacy and health outcomes must be interpreted with caution. A further study examining the point that the likelihood of mortality was high in the polypharmacy group might be attributed to adverse drug events of polypharmacy will be necessary.

In conclusion, this study demonstrates the association between polypharmacy and adverse health outcomes such as hospitalization, emergency department visits, and mortality, in a large population-based study. We found that polypharmacy was associated with adverse health outcomes. However, further studies that are larger and multinational in scope are needed to confirm these results.

## Data Availability

The raw data supporting the conclusions of this article will be made available by the authors, without undue reservation.
